# Cortical Reorganization following Injury Early in Life

**DOI:** 10.1155/2016/8615872

**Published:** 2016-05-19

**Authors:** Moran Artzi, Shelly Irene Shiran, Maya Weinstein, Vicki Myers, Ricardo Tarrasch, Mitchell Schertz, Aviva Fattal-Valevski, Elka Miller, Andrew M. Gordon, Dido Green, Dafna Ben Bashat

**Affiliations:** ^1^Functional Brain Center, The Wohl Institute for Advanced Imaging, Tel Aviv Sourasky Medical Center, 6 Weizmann Street, 64239 Tel Aviv, Israel; ^2^Sackler Faculty of Medicine, Tel Aviv University, Tel Aviv, Israel; ^3^Department of Radiology, Tel Aviv Sourasky Medical Center, 6 Weizmann Street, 64239 Tel Aviv, Israel; ^4^Gonda Multidisciplinary Brain Research Center, Bar Ilan University, Bar Ilan, Israel; ^5^Jaime and Joan Constantiner School of Education, Tel Aviv University, P.O. Box 39040, 69978 Tel Aviv, Israel; ^6^Sagol School of Neuroscience, Tel Aviv University, Tel Aviv, Israel; ^7^Paediatric Neurology Unit, Tel Aviv Sourasky Medical Center, 6 Weizmann Street, 64239 Tel Aviv, Israel; ^8^Diagnostic Imaging, Children's Hospital of Eastern Ontario, 401 Smyth Road Ottawa, ON, Canada K1H 8L1; ^9^Department of Biobehavioral Sciences, Teachers College, Columbia University, 525 W 120th Street, New York, NY 10027, USA; ^10^Department Occupational Therapy, Faculty of Medicine, Tel Aviv University, Tel Aviv, Israel; ^11^Centre for Rehabilitation, Oxford Brookes University, Headington Campus, Headington Road, Oxford OX3 0BP, UK

## Abstract

The brain has a remarkable capacity for reorganization following injury, especially during the first years of life. Knowledge of structural reorganization and its consequences following perinatal injury is sparse. Here we studied changes in brain tissue volume, morphology, perfusion, and integrity in children with hemiplegia compared to typically developing children, using MRI. Children with hemiplegia demonstrated reduced total cerebral volume, with increased cerebrospinal fluid (CSF) and reduced total white matter volumes, with no differences in total gray matter volume, compared to typically developing children. An increase in cortical thickness at the hemisphere contralateral to the lesion (CLH) was detected in motor and language areas, which may reflect compensation for the gray matter loss in the lesion area or retention of ipsilateral pathways. In addition, reduced cortical thickness, perfusion, and surface area were detected in limbic areas. Increased CSF volume and precentral cortical thickness and reduced white matter volume were correlated with worse motor performance. Brain reorganization of the gray matter within the CLH, while not necessarily indicating better outcome, is suggested as a response to neuronal deficits following injury early in life.

## 1. Introduction

During gestation and up to 2 years of age, the human brain develops at an astounding rate. While brain injury during this critical period can have devastating consequences, the brain has a remarkable capacity for reorganization following perinatal injury [1–6]. One of the most common disorders is cerebral palsy, which arises in the early developing fetal or infant brain in 1-2/1000 children in Western countries [7]. This type of brain injury is characterized by mild to severe motor impairment of unilateral (hemiplegia) or bilateral (diplegia or quadriplegia) distribution and can present with global physical and mental dysfunction.

Magnetic resonance imaging (MRI) is considered the gold standard for structural and functional imaging in many brain pathologies including cerebral palsy [3, 8, 9]. Imaging findings following brain injury early in life demonstrate an increase in cerebrospinal fluid (CSF) volume and reduced gray matter (GM) and white matter (WM) volumes [1, 10–12], with the latter being the most common imaging finding in children with hemiplegia [9, 10, 13, 14].

Brain reorganization and plasticity in children with cerebral palsy following early injury and after various interventional programs has been shown using several modalities including visual and somatosensory evoked potentials (VEPs), electroencephalogram (EEG), and MRI [15]. Such brain changes have been observed not only in the vicinity of the injured area but also in distant areas including the area within the contralateral hemisphere (CLH), contralateral to the injury. Evidence of brain reorganization has been detected in the ipsilesional sensory, motor, and language areas, based on diffusion tensor imaging (DTI) and functional MRI (fMRI) [1, 12, 16–23] and hypertrophy, detected in the corticospinal tracts (CST) in the noninfarcted hemisphere [24]. However, knowledge regarding the effect of such changes on behavior and whether recruitment of brain areas in the contralateral hemisphere improves outcome is limited.

While several neuroimaging studies have shown a correlation between lesion size and neurobehavioral abilities in children with hemiplegia [14, 16, 25–27], others found that lesion size does not always correlate with behavioral performance [28, 29]. Several studies showed that the size of the lesion and the age of the injury affect the patterns of brain activation [30, 31]. However, the effects of timing and lesion size on brain reorganization as mediators for neurobehavioral performance remains open to debate.

The aim of this work was to study brain changes following injury early in life in children with hemiplegia, compared to typically developing children (TDC), and to assess the correlation between structural changes and motor performance. We studied brain reorganization in the CLH in relation to the size of the injury, hypothesizing that greater reorganization would be evident in children with larger volume of tissue loss. We focused on changes in GM and WM, using measures of volume, morphology, integrity, and perfusion.

## 2. Methods

### 2.1. Participants

Fifteen children with hemiplegia (eight females, mean age 12.5 ± 3.0 years, 14 with perinatal injury and one child with injury at the age of 3 months), from a cohort attending for assessment prior to participation in an intensive motor therapy program [32], and sixteen age- and gender-matched TDC (nine males, mean age 10.8 ± 2.9 years) were included in this study. Children with hemiplegia were recruited from a regional hospital and/or child development center. Children were included in the study if they had cerebral palsy with clinical signs of spastic hemiplegia, were attending regular education, and were independently mobile. Exclusion criteria were any overt seizure activity, treatment to improve motion in the prior six months, and any contraindications to MRI. The control group included TDC attending an age-appropriate educational facility, with no brain anomalies on conventional MRI, no prior history of head injury, and no clinical evidence of neurological dysfunction. The study was approved by the review board of the Ministry of Health and the institutional review board of the hospital. Parents or legal guardians of the participants and the children provided informed consent.

### 2.2. Imaging

MRI scans were performed on a 3.0 T MRI scanner (GE Signa Excite, Milwaukee, WI, USA) using an eight-channel head coil. MRI protocol included three-dimensional (3D) high-resolution anatomical *T*_1_-weighted fast spoiled gradient echo (FSPGR) imaging (field of view (FOV)/matrix = 256 mm^2^/256 × 256, repetition time (*TR*)/echo time (*TE*) = 8.6/3.3 ms); gradient echo *T*_2_^*∗*^ (GRE *T*_2_^*∗*^) (FOV/matrix = 240 mm^2^/512 × 512, *TR*/*TE* = 320/20 ms); perfusion imaging performed using 3D pseudocontinuous arterial spin labeling (ASL) (FOV/matrix = 240 mm^2^/128 × 128, *TR*/*TE* = 4580/9.8 ms, postlabeling  delay = 1500 ms); and DTI acquired along 19 diffusion gradient directions (*b* = 1000 mm^2^/sec) and one with no applied diffusion gradient (FOV/matrix = 220 mm^2^/128 × 128, *TR*/*TE* = 11,000/91 ms).

### 2.3. Image Analysis

First, total cerebrum volume of GM and WM was assessed in each subject and compared between groups. All other imaging parameters were measured separately in the right and left hemispheres in the TDC and only in the CLH in the children with hemiplegia.

**Figure 1:**
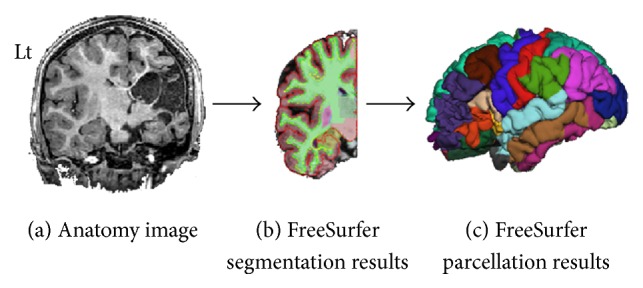
Parcellation of the hemisphere contralateral (CLH) to the lesion in a 7-year-old female with right lesion. (a) High-resolution anatomical 3D *T*_1_ W image; (b) FreeSurfer segmentation results in the CLH; (c) 3D visualization of cortical parcellation obtained for the left hemisphere.

Within the CLH, volumetric and morphometric measurements were calculated from high-resolution 3D *T*_1_-weighted anatomical images; cerebral blood flow (CBF) was assessed using ASL; and tissue integrity, mean diffusivity, and fractional anisotropy (MD and FA) values were measured using DTI. These imaging parameters were studied within the segmented cerebral GM and WM areas, and within 35 cortical areas defined based on FreeSurfer anatomical segmentation [33, 34] for both hemispheres in the TDC and only for the CLH in children with hemiplegia (Figure 1).

*Preprocessing*. In each subject, all images and calculated maps were realigned into the GRE *T*_2_^*∗*^ images using FMRIB Software Library (FSL) linear image registration tool [35]. Brain extraction was performed using FSL brain extraction tool. Inhomogeneity correction was performed on the anatomical *T*_1_-weighted images, using N3 MINC B0 (part of FreeSurfer, v. 4.0.5).

*Brain Tissue Segmentation*. Segmentation into CSF, GM, and WM was performed on the *T*_1_-weighted 3D high-resolution anatomical images separately for each subject, using FSL automatic segmentation tool [36], with the number of clusters (*k*) = 3. Volumes of each cluster (identified as CSF/GM/WM) were calculated in percentages relative to the entire brain volume of each subject. The cerebellum and brain stem were excluded from all images and calculated maps using anatomical masks obtained based on Harvard-Oxford cortical and subcortical structural atlases (part of FSL).

*Cortical Parcellation*. Cortical reconstruction and volumetric segmentation were performed on the *T*_1_-weighted 3D high-resolution anatomical images using the FreeSurfer analysis tools (http://surfer.nmr.mgh.harvard.edu/). Briefly, this processing includes brain extraction, automated Talairach transformation, and segmentation of the subcortical WM and deep GM volumetric structures (including hippocampus, amygdala, caudate, putamen, and ventricles) [34]. Mean values of thickness (mm) and area (mm^2^) were measured in 35 cortical areas based on the Desikan-Killiany Atlas [33, 34]. The children with hemiplegia included in this study had varying degrees of brain injury that in some cases prevented accurate alignment to a standard space. In these cases (*n* = 4) the anatomical images were modified by replacing the affected hemisphere with the CLH (just for the purpose of registration).

*Perfusion Analysis. *Cerebral blood flow (CBF) maps were calculated from the ASL data based on Järnum et al. [37] using the following equation:

(1) $$\begin{array}{c} f=\frac{\lambda }{2\alpha T_{1b}\left(1-e^{-\tau /T_{1b}}\right)}\frac{\left(S_{ctrl}-S_{lbl}\right)\left(1-e^{-t_{sat}/T_{1g}}\right)}{S_{ref}}e\frac{w}{1b}, \end{array}$$

where *f* is flow (mL/min/100 g); *λ* = 0.9 is the brain–blood partition coefficient [37]; (*S*_ctrl_ − *S*_lbl_) is the ASL control (*S*_ctrl_) − labeled (*S*_lbl_) images; *t*_sat_ = 2,000 ms is the correction for the incomplete recovery due to the saturation performed before imaging [37]; *T*_1*g*_ = 1421 ms is *T*_1_ of the GM [38]; *α* = 0.8 is the labeling efficiency [37]; *T*_1*b*_ = 1,600 ms is *T*_1_ of the blood [25, 38]; *τ* = 1525 ms is labeling duration; *S*_ref_ = ASL is reference proton density images; *w* = 1500 ms is the postlabeling delay time. 

*DTI Analysis. *Mean diffusivity (MD) and fractional anisotropy (FA) maps were calculated from the DTI data using FSL diffusion tool; mean MD and FA values where calculated within the WM, and only voxels with FA values >0.2 were included (in order to minimize partial volume effects). Within the GM areas, only MD values were calculated.

CBF maps (in mL/min/100 g) and MD values were measured within the 35 cortical regions and were compared between groups.

### 2.4. Neurobehavioral Assessment

Neurobehavioral assessment included the assisting hand assessment (AHA, version 4.3), for evaluation of how children with hemiplegia spontaneously use the affected hand in bimanual play; higher scores represent better bimanual skills [39]; the Children's Hand Experience Questionnaire (CHEQ) was used for exploration of the independent participation and skilled use of an affected/hemiplegic hand in daily bimanual activities Sköld et al. [40]. Two measures were used: CHEQ% use, the extent to which the child's affected hand was used in daily bimanual activities, calculated as a percentage of independent activities (the affected hand was used to stabilise or grip items), and CHEQ 2 hands, the number of activities performed using two hands. In addition, the Jebsen Taylor Test of Hand Function was used (JTTHF; [41]), documenting efficiency (timed in seconds) of a range of grips and ability to release items, with higher values reflecting slower, worse ability. A normalized ratio score for the JTTHF was also calculated to represent the relative balance of motor ability between the affected hand and the less affected hand [JTTHF ratio score = (JTTHF score using the affected hand − JTTHF score using the less affected hand)/(JTTHF score using the affected hand + JTTHF score using the less affected hand)], values ranged from 0 to 1 [42].

## 3. Statistical Analysis

Statistical analysis was performed using SPSS (SPSS Inc., Chicago, IL, USA). Paired sample* t*-tests were used to compare MRI parameters in the right and left hemispheres in the TDC. Between groups comparisons were performed for the homological hemisphere (as in the TDC group, significant differences were detected between hemispheres for several MRI parameters). As three children had left hemiplegia and 12 had right hemiplegia, all values were standardized relative to the mean value of the TDC group, calculated separately for each hemisphere, and comparisons were performed for the homological hemisphere in the control group while maintaining the proportion of right to left hemisphere lesions found in the children with hemiplegia group (12 : 3). For each parameter we randomly chose data from the left hemisphere from 4 children in the control group, by randomized selection of the obtained value, using Research Randomizer tool (version 3.0) [43].

Since we hypothesized that the degree of brain reorganization would be dependent on the extent of injury, children with hemiplegia were divided into two subgroups based on their CSF volume (indirect measure of brain tissue lost); children with small lesions (SL) in which the CSF volume was within two standard deviations (SD) of that of the TDC and children with large lesions (LL) with CSF volume > 2 SD of the TDC. One way ANOVA with Bonferroni correction for multiple comparisons with *p* ≤ 0.01 was used to compare the three groups (TDC, SL, and LL) for all MRI parameters. Spearman's correlation was performed between the MRI parameters and behavioral assessment.

## 4. Results

Of the fifteen children with hemiplegia included in the study, 12 had right hemiplegia (left hemispheric injury) and three had left hemiplegia (right hemispheric injury). Sixteen TDC served as a control group. No significant age and gender differences were detected between groups. No child had aphasia and normal speech was reported for all children. Characteristics of children with hemiplegia including perinatal factors and neurobehavioral assessment results are summarized in Table 1.

### 4.1. Changes in Total Cerebral Tissue Volume

**Figure 2:**
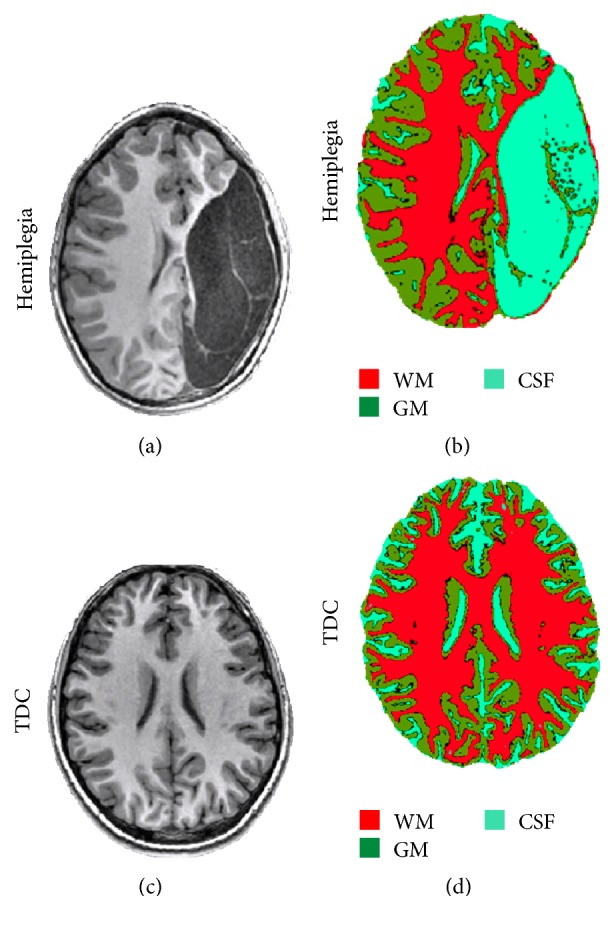
Representative data demonstrating brain anatomy images and cerebral tissue segmentation results obtained from a 7-year-old child with hemiplegia (a-b) and from a healthy age-matched control (c-d); the three obtained clusters were identified as white matter (WM), gray matter (GM), and cerebrospinal fluid (CSF). Note the increased cortical thickness in the hemisphere contralateral to the lesion of the children with hemiplegia (b) compared to TDC (d).

The cerebral hemispheres of each subject were segmented into CSF, GM, and WM using an unsupervised segmentation algorithm. Significant (*p* < 0.005) increased CSF volume (in percentages relative to the total cerebral volume) was detected in the study group (19 ± 5%) compared to TDC (14 ± 1%), with a significant (*p* < 0.001) reduction in total WM volume (33 ± 3% in the study group and 37 ± 2% in TDC), with no significant difference in total GM volume (42 ± 4% in the study group and 42 ± 2% in TDC).  Figure 2 shows representative anatomic and segmentation results obtained from a seven-year-old child with hemiplegia (Figures 2(a) and 2(b)) and from an age-matched TDC (Figures 2(c) and 2(d)).

*Correlations with Behavioral Assessments*. The CSF volume was negatively correlated with AHA score (*r* = −0.53, *p* = 0.04) and CHEQ% use (*r* = −0.63, *p* = 0.01) and positively correlated with the unimanual capacity of the affected hand on the JTTHF (*r* = 0.70, *p* = 0.004) and with the JTTHF ratio score (*r* = 0.62, *p* = 0.02). The WM volume was negatively correlated with the unimanual capacity of the affected hand on the JTTHF (*r* = −0.70, *p* = 0.003) and positively correlated with the CHEQ 2 hands (*r* = 0.65, *p* = 0.008) and with CHEQ% use (*r* = 0.66, *p* = 0.008). A high correlation was detected between JTTHF ratio score and JTTHF score of the affected hand (*r* = 0.89, *p* < 0.0001); therefore, all results indicate that worse performance is associated with increased CSF volume and reduced WM volume, that is, larger extent of injury.

Seven children were classified in the SL group, with CSF volume within two SD of that of the TDC group (mean ± SD volume of the CSF in TDC = 14 ± 2%, and in the SL group = 15 ± 1%, percent of total cerebral volume), and eight children were classified in the LL group, with CSF volume >2 SD of that of the TDC group (mean ± SD  22 ± 4% total cerebral volume). We further assessed differences between groups only in the CLH, due to methodological issues (see Methods and Discussion).

### 4.2. Gray Matter Changes within the CLH

*Volumetric Changes*. A significant group difference (*p* < 0.001) was detected with increased GM volume in the LL group compared to TDC. There was no significant difference in GM volume between the SL group and either LL group or TDC. 

**Figure 3:**
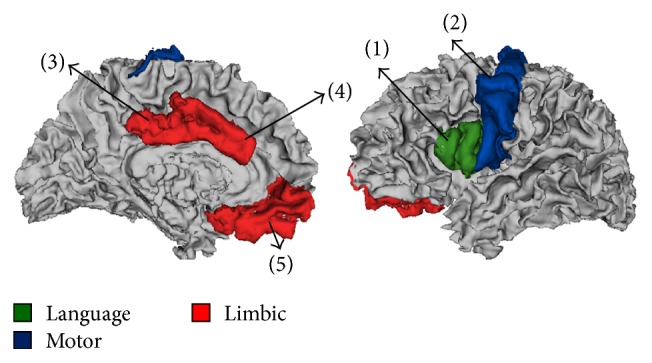
Anatomical locations of cortical brain areas in which significant group differences were detected for the cortical thickness, surface area, and CBF parameters; language area (green); motor area (blue); and limbic area (red). (1) Pars opercularis; (2) precentral; (3) posterior cingulated; (4) caudal anterior cingulate; and (5) medial orbitofrontal.

**Figure 4:**
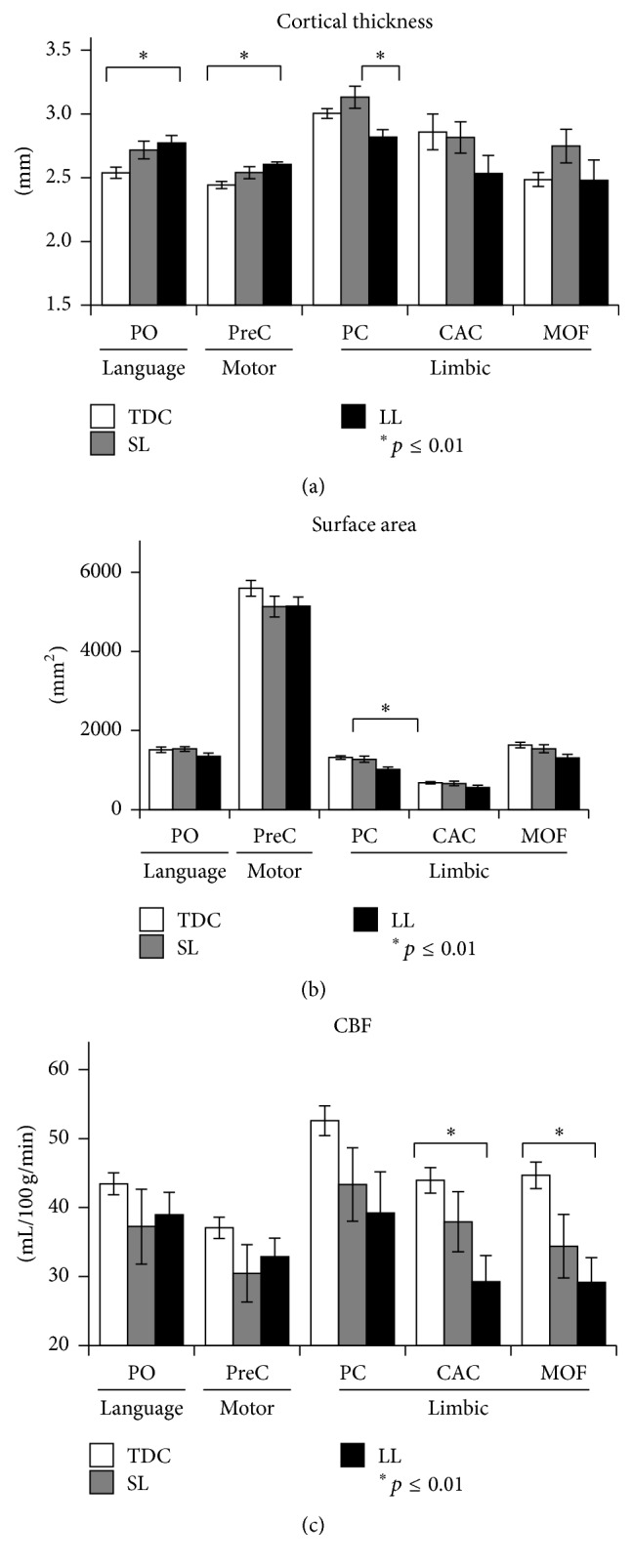
Means and standard deviations of cortical brain areas in which significant group differences (*p* ≤ 0.01) for cortical thickness (a), surface area (b), and cerebral blood flow (CBF) (c) were detected. The mean values are shown for the various anatomical locations of the cortical brain areas: motor area; language area; and limbic area and for the three different groups: control, small lesion (SL), and for the large lesion group (LL). PO: pars opercularis; PreC: precentral; PC: posterior cingulate; CAC: caudal anterior cingulate; MOF: medial orbitofrontal.

*Cortical Morphology Analysis. *Significant group differences were detected in several cortical areas located within three functional networks: language, motor, and limbic areas (Figure 3).  Figure 4 shows mean values of cortical thickness and surface area, obtained in cortical areas in which significant group differences were detected. Cortical thickness: significantly increased cortical thickness (*p* < 0.01) was detected in the LL group compared to TDC in language area (pars opercularis (PO)) and motor area (precentral (PreC)). Significantly reduced cortical thickness was detected in the LL group compared to the SL group in the limbic area (posterior cingulated (PC)). Cortical surface area: significantly reduced surface area (*p* < 0.01) was detected in the LL group compared to the TDC only in the limbic area (PC).

Overall, the increased cortical thickness, detected in several cortical areas, in LL group compared with TDC, can also be seen by visual inspection of the anatomical images (Figure 2) and may be explained as compensation for GM loss in the injured hemisphere, without additional sulcation, resulting in preserved cortical surface in areas with increased cortical thickness. 

*Cortical Perfusion.* ASL perfusion imaging was performed in all TDC (*n* = 16) and in eight children with hemiplegia, of which three were classified within the SL group and five within the LL group. Significant group differences were found (*p* < 0.01), with reduced CBF values detected in the LL group compared to TDC in limbic areas (caudal anterior (CAC) and medial orbitofrontal (MOF)) (Figure 4(c)). No significant increase in CBF values was detected in children with hemiplegia compared to TDC and no differences were detected between the SL group and LL or TDC.

*Cortical Diffusion.* DTI was performed in nine TDC and in all children with hemiplegia (*n* = 15). Significant group differences were detected for the MD values, with increased values (indicating reduced integrity), in the LL group compared to TDC in the sensory-motor (postcentral, paracentral, and superior parietal), language (superior temporal and pars orbitalis), limbic (rostral anterior cingulate, isthmus cingulate, posterior cingulate, and medial orbitofrontal), cognitive-memory (entorhinal and precuneus), auditory (transverse temporal), and visual areas (cuneus, pericalcarine, lateral occipital, and lingual). Significantly increased MD values were also detected in the LL group compared to the SL group, within the superior parietal, medial orbitofrontal, isthmus cingulate, precuneus, cuneus, and lateral occipital areas. No reductions in MD values were detected in children with hemiplegia compared to TDC in all cortical regions.

*Correlation between Gray Matter Changes and Behavioral Assessments*. Since motor tasks were assessed in the children with hemiplegia, correlations were analyzed only for MRI parameters in the corresponding precentral area (in which significant differences were detected between groups). Significant positive correlation was detected between cortical thickness in the precentral areas and the JTTHF ratio score (*r* = 0.6, *p* = 0.02), suggesting that worse performance is associated with increased cortical thickness. This was also supported by a borderline negative correlation (*r* = −0.46, *p* = 0.08) detected between cortical thickness in the precentral areas and CHEQ% use. Behavioral assessments did not correlate significantly with CBF or tissue integrity values in none of the segmented area.

### 4.3. WM Changes

No significant group differences were detected for all MRI parameters in the WM, including the CBF values (TDC = 30.6 ± 3.7, SL = 25.6 ± 5.6, LL = 27.6 ± 3.5 (mL/min/100 g)); the MD values (TDC = 0.86 ± 0.03, SL = 0.88 ± 0.03, LL = 0.91 ± 0.03 (mm/sec^2^ × 10^−3^)), or the FA values (TDC = 0.44 ± 0.02, SL = 0.43 ± 0.02, LL = 0.41 ± 0.02 (arbitrary units)).

## 5. Discussion

This study characterizes brain reorganization following early brain injury in children with hemiplegia. Our results show that GM volume was preserved even in cases of large lesions. Compensation for the GM loss in the injured area was attained by increased GM thickness in language and motor areas, in the CLH, yet with no changes in surface area or perfusion in these areas. Reduced cortical thickness, surface area, and perfusion were detected in limbic areas. The total WM volume was significantly reduced, accompanied by increased CSF volume, with no increase in WM volume within the CLH.

As previously reported, the extent of tissue plasticity depends on the age of the injury and the size of the lesion [30, 31]. Moreover, the location of the lesion, related to different functional areas, and hemispheric lateralization may also affect brain reorganization. In this study, all children sustained injury early in life, 14 perinatally and one child at 3 months of age. Children who had injury later in life were not included in this study, as time of injury may influence plasticity processes. Furthermore, early in development there may be a critical period in which the brain possesses high capacity for reorganization to compensate for injury and the extent of this period is still unknown. Regarding the size of the lesion, we divided the children with hemiplegia into two subgroups, based on their lesion size, as indirectly measured by the total CSF volume. Substantial brain differences were found in both the macro- and microstructure levels between children with mild to severe hemiplegia and controls.

Evidence of structural and functional brain reorganization was previously demonstrated using various imaging techniques including DTI, Transcranial Magnetic Stimulation [12, 16], or fMRI [17–20, 24]. In the current work, quantitative analyses of morphometry, perfusion, and tissue integrity parameters were performed in children with mild to severe injury. Recruitment of brain areas distant to the lesion, including within the unaffected hemisphere, have been mainly studied following stroke [44]. Our results showed that the GM loss in children with hemiplegia was corresponded to increased cortical thickness within the CLH in motor and language areas. These results support previous findings following brain injury early in life (which used other methods) detecting changes predominantly in motor and language areas [1, 6, 19, 21].

Previous studies in patients with chronic injury suggested that recovery of motor function relies predominantly on the extent and location of brain reorganization and that not all forms of neural plasticities contribute to genuine motor recovery. In general, it is suggested that less efficient recovery may be obtained when compensation is manifested in the contralesional hemisphere, rather than in the injured hemisphere. Physiological changes in patients following stroke in the CLH were shown to produce a less efficient recovery than physiological changes in the damaged hemisphere [45]. Reduced linguistic performance was detected in children following childhood insults, when the increased activation was detected in the CLH (nondominant) [46]. Results obtained in our previous fMRI study in this cohort showed that better hand function was associated with more activation in primary motor area of the affected hemisphere [32] and also that improvement in hand function following intervention was associated with increased level of activation in the affected hemisphere (a shift to a more unilateral activation) [47]. Based on our findings, in conjunction with previous evidence, it is suggested that brain reorganization within the CLH in children with TDC hemiplegia is not necessarily associated with better prognosis but on the contrary may even adversely affect recovery and intervention outcome. The current study showed initial results regarding correlation with brain reorganization in the CLH and motor function but was unable to map brain reorganization in the affected hemisphere. Further studies should assess if perilesional, rather than contralesional, reorganization is more efficient, resulting in better outcomes and whether it may predict recovery following intervention.

Reduced total WM volume was detected in children with hemiplegia compared to TDC. This finding is consistent with previous studies in which WM injury was found to be the most common imaging finding in children with hemiplegia/cerebral palsy [8–10, 48, 49]. Reduced WM volume in the affected hemisphere was not compensated by increased volume in the CLH as was the case for GM. Although previous DTI studies reported WM reorganization, indicated by increased number of fibers and FA values in the unaffected hemisphere in children with hemiplegia [16] and demonstrating reorganization in the CST in the noninfarcted hemisphere associated with worse hand function [17, 24], the current study and our previous work [32] showed no differences in tissue integrity and/or perfusion of the WM in the CLH in our cohort compared to TDC.

Regarding vascular changes, previous studies investigating brain perfusion in children with hemiplegia, including MRI methods and single photon emission computer tomography, reported contradictory results, which may in part have been due to variations in the etiologies of hemiplegia within and between studies [50–53]. In this study, cortical perfusion measurements revealed significantly reduced CBF values in limbic areas, compared to TDC. In motor and language areas, no significant differences were detected between groups. This finding might suggest an increase in the cerebral blood volume, to support the increased GM thickness. However, in this study this parameter was not available, limiting our ability to draw reliable conclusions.

Despite the importance of studying children with large lesions, which is vital to improve our understanding of brain reorganization during development, both in children with hemiplegia and in other developmental pathologies, most previous studies excluded this group due to methodological difficulties. Volumetric assessment of brain tissue necessitates segmentation, and commonly used segmentation methods rely on differences in signal intensity and integration of prior information such as tissue probability maps and/or require realignment of the subject to a standard space. This precludes their use in cases with substantial brain deformation, and most previously reported studies of subjects with hemiplegia therefore either excluded subjects with large lesions or avoided such analyses. In the current study, we overcame this problem using two different approaches: the first used an unsupervised segmentation algorithm that enabled segmentation and quantification of tissue volumes within the entire brain (global). This method requires neither prior information nor data normalization and thus is most suitable for the study of various populations with severe brain deformation. Next, we studied local changes by modifying the brain in the LL group. This procedure enabled us to realign the brain to a standard space and reliably study cortical changes but only within the CLH.

Several limitations of this study should be considered: due to the unique study population, the number of subjects is relatively small, which prevents us from drawing generalized conclusions. Yet, our study group is relatively homogenous, including only subjects following perinatal injury and with an age/gender-matched TDC. The lack of quantitative structural and vascular assessment of the affected hemisphere, due to severe brain deformation, prevents comparison of brain reorganization between the CLH and the affected hemisphere and how this affects performance. Strengths of this study include inclusion of subjects with moderate-severe injury, enabling a better understanding of brain reorganization in this population, quantitative assessment of brain morphometry, and tissue integrity and vascularity in this unique group.

## 6. Conclusion

This study provides evidence and characterization of the cerebral reorganization which occurs following injury early in life. GM reorganization, related to the time of the insult, extent, and anatomical location, is suggested to play a pivotal role in neurobehavioral performance and may serve as a biomarker for intervention guidance.

## Figures and Tables

**Table 1:** Subject characteristics.

Number	Gender	Age	Hemiparetic side	Type of injury	MACS
1	F	14	R	PVL/IVH	3
2	M	8	R	Infarct/contusion	3
3	F	14	R	PVL/IVH	2
4	F	13	R	PVL/IVH	2
5	M	11	L	PVL/IVH	1
6	M	14	R	PVL/IVH	1
7	M	9	R	Infract/contusion	3
8	F	10	R	PVL/IVH	1
9	F	16	R		1
10	M	9	R	PVL/IVH	2
11	F	7	R	Infarct/contusion	2
12	M	7	L	Infarct/contusion	1
13	M	8	R	PVL/IVH	3
14	F	10	R	PVL/IVH	2
15	F	12	L	Infarct/contusion	2

MACS (severity of hemiparesis): manual ability classification system; PVL: periventricular leukomalacia; IVH: intraventricular hemorrhage.

## Abbreviations

CSF: Cerebrospinal fluid
CLH: Contralateral hemisphere to the injury
MRI: Magnetic resonance imaging
GM: Gray matter
WM: White matter
VEPs: Evoked potentials
EEG: Electroencephalogram
DTI: Diffusion tensor imaging
fMRI: Functional MRI
CST: Corticospinal tracts
TDC: Typically developing children
FSPGR: Fast spoiled gradient echo
*TR*: Repetition time
*TE*: Echo time
FOV: Field of view
GRE: Gradient echo
ASL: Arterial spin labeling
CBF: Cerebral blood flow
MD: Mean diffusivity
FA: Fractional anisotropy
CHEQ: Children's hand experience questionnaire
JTTHF: Jebsen Taylor Test of hand function
SD: Standard deviations
SL: Small lesions
LL: Large lesions
MACS: Manual ability classification system
PVL: Periventricular leukomalacia
IVH: Intraventricular hemorrhage
CON: Control
PO: Pars opercularis
PreC: Precentral
PC: Posterior cingulate
CAC: Caudal anterior cingulate
MOF: Medial orbitofrontal.
